# Estimating Brain Functional Networks Based on Adaptively-Weighted fMRI Signals for MCI Identification

**DOI:** 10.3389/fnagi.2020.595322

**Published:** 2021-01-14

**Authors:** Huihui Chen, Yining Zhang, Limei Zhang, Lishan Qiao, Dinggang Shen

**Affiliations:** ^1^School of Mathematics Science, Liaocheng University, Liaocheng, China; ^2^Department of Research and Development, Shanghai United Imaging Intelligence Co. Ltd., Shanghai, China; ^3^Department of Brain and Cognitive Engineering, Korea University, Seoul, South Korea

**Keywords:** index terms-brain functional network, functional magnetic resonance imaging, scrubbing, Pearson's correlation, sparse re presentation, mild cognitive impairment

## Abstract

Brain functional network (BFN) analysis is becoming a crucial way to explore the inherent organized pattern of the brain and reveal potential biomarkers for diagnosing neurological or psychological disorders. In so doing, a well-estimated BFN is of great concern. In practice, however, noises or artifacts involved in the observed data (i.e., fMRI time series in this paper) generally lead to a poor estimation of BFN, and thus a complex preprocessing pipeline is often used to improve the quality of the data prior to BFN estimation. One of the popular preprocessing steps is data-scrubbing that aims at removing “bad” volumes from the fMRI time series according to the amplitude of the head motion. Despite its helpfulness in general, this traditional scrubbing scheme cannot guarantee that the removed volumes are necessarily unhelpful, since such a step is fully independent to the subsequent BFN estimation task. Moreover, the removal of volumes would reduce the statistical power, and different numbers of volumes are generally scrubbed for different subjects, resulting in an inconsistency or bias in the estimated BFNs. To address these issues, we develop a new learning framework that conducts BFN estimation and data-scrubbing simultaneously by an alternating optimization algorithm. The newly developed algorithm adaptively weights volumes (instead of removing them directly) for the task of BFN estimation. As a result, the proposed method can not only reduce the difficulty of threshold selection involved in the traditional scrubbing scheme, but also provide a more flexible framework that scrubs the data in the subsequent FBN estimation model. Finally, we validate the proposed method by identifying subjects with mild cognitive impairment (MCI) from normal controls based on the estimated BFNs, achieving an 80.22% classification accuracy, which significantly improves the baseline methods.

## Introduction

Functional magnetic resonance imaging (fMRI), by detecting the change of cerebral blood oxygen saturation degree, achieves the purpose of non-invasive “observation” of the brain activities (Brunetti et al., 2006; Whittingstall et al., 2008). Based on the observed brain activities in fMRI, one can explore the brain *in vivo* from multiple aspects. For example, in 1995, Biswal et al. first showed activation maps of the human brain by fMRI data, under a state in which the subject did not carry out any specific tasks (Biswal et al., 1995), which opened a new area for studying spontaneous fluctuations of the brain at resting state.

While resting-state fMRI (rs-fMRI) is potentially useful in clinical practice, finding biomarkers that can identify patients from normal controls (NCs) has been a primary driver of resting state research over the last decades (Li et al., 2019b). Unfortunately, it is hard to reveal informative patterns by a direct comparison of the fMRI time series between different subjects, since the fMRI signals are arbitrarily scaled and have no unit (Jenkinson and Chappell, 2018). In contrast, brain functional network (BFN), as a measure of the relative relationship between the fMRI time series, can provide a more reliable way of exploring the inherent organization of the brain (Liu et al., 2015; Yu et al., 2019), and has been used in identifying neurological or psychiatric disorders (Stam, 2014), including autism spectrum disorder (Weikai et al., 2017), major depressive disorder (Greicius et al., 2007), obsessive compulsive disorder (Admon et al., 2012), Alzheimer's disease (AD) (Jin et al., 2010; Shi et al., 2017), and its early stage, namely mild cognitive impairment (MCI) (Yu et al., 2017; Li et al., 2019b), to name a few.

Due to its importance, researchers have proposed a series of BFN estimation methods in recent years. However, in general, estimating a “good” BFN is still an extremely challenging problem, because complex artifacts or noises are always involved in the observed fMRI data. In practice, a preprocessing pipeline, including motion correction, nuisance regression, spatial smoothing and temporal filtering (Poldrack et al., 2011), etc., is employed to improve the quality of data. For example, as a simple and popular preprocessing step, the scrubbing operation has been investigated to clean potentially “bad” volumes if the head motion measured by frame-wise displacement (FD) (Power et al., 2012a) or DVARS (Yan et al., 2013) is greater than a threshold. Despite its helpfulness in general, it is hard to guarantee that all the removed volumes are necessarily unhelpful, since the scrubbing operation is independent of the ensuing BFN estimation task.

Recently, Li et al. proposed a task-dependent scrubbing method by incorporating the scrubbing operation into the BFN estimation task (Li et al., 2019a). Although it can optimize the data scrubbing and BFN estimation jointly, Li's method removes the “bad” volumes by a *hard* regularized parameter that is similar to the thresholding scheme used in the traditional scrubbing strategy. As a result, different numbers of volumes are generally removed for different subjects, which leads to a bias or inconsistency in the estimated BFNs. Moreover, the hard removal of volumes always reduces the statistical power, which is a serious problem, especially when the number of volumes is limited in the fMRI time series.

To address these issues, in this paper, we develop a new learning approach for BFN estimation based on weighted fMRI time series, where the weight on each volume is adaptively estimated from the data. Consequently, the proposed method not only reduces the difficulty of threshold selection involved in the traditional scrubbing scheme, but also provides a framework for a more flexible data “scrubbing” operation that jointly works with the BFN estimation task. Finally, we validate the proposed method by using it to identify subjects with MCI from NCs based on a publicly available dataset. The experimental results illustrate that the proposed approach can achieve better classification accuracy than the baseline methods. For reproducing our results, we also share the source codes in https://github.com/Huihui-Chen/Adaptively-weighted.

The rest of this paper is organized as follows. In Section related methods, we review two baseline methods, PC and SR, for BFN estimation. In Section BFN estimation, we propose the new BFN learning framework, including its motivation, model, and algorithm. In Section experiments, we evaluate our proposed method with experiments. Finally, we conclude the paper with a brief discussion in Section conclusion.

## Related Methods

Due to the potential applications in exploring the inherent organization and neurodegenerative diseases of the brain, many BFN estimation methods have been proposed in the past decades. In this section, we review two representatives of them, PC and SR, which are closely related to our study.

### Pearson's Correlation

Suppose that the brain, according to a certain atlas, has been parcellated into *N* regions of interest (ROIs), and there are *T* measurements over time, each of the ROIs correspond to an extracted time series ${\text{x}}_{i}\in R^{T\times 1}$, *i* = 1, ⋯ , *N*. Then, the edge weight matrix **C**=(_*C*_*ij*_)*N* × *N*_ of PC-based BFN can be defined as follows:

(1) $$\begin{array}{l} C_{ij}=\frac{{\left(x_{i}-{\bar{x}}_{i}\right)}^{T}(x_{j}-{\bar{x}}_{j})}{\sqrt{{\left(x_{i}-{\bar{x}}_{i}\right)}^{T}(x_{i}-{\bar{x}}_{i})}\sqrt{{\left(x_{j}-{\bar{x}}_{j}\right)}^{T}(x_{j}-{\bar{x}}_{j})}} \end{array}$$

Where *C*_*ij*_ is the estimated functional connectivity between the *i*th and *j*th ROIs, and ${\overset{-}{\text{x}}}_{i}$ is the mean vector corresponding to **x**_*i*_. Without loss of generality, we define a new ${\text{x}}_{i}≜\left({\text{x}}_{i}-{\overset{-}{\text{x}}}_{i}\right)/\sqrt{{\left({\text{x}}_{i}-{\overset{-}{\text{x}}}_{i}\right)}^{T}\left({\text{x}}_{i}-{\overset{-}{\text{x}}}_{i}\right)}$. As a result, **Equation (1)** can be simplified into $C_{ij}={{\text{x}}_{i}}^{T}{\text{x}}_{j}$. Further, we suppose $\text{X}=\left[{\text{x}}_{1},{\text{x}}_{2},\cdots \;,{\text{x}}_{N}\right]\in R^{T\times N}$, and thus we have **C** = **X**^*T*^**X**. Equivalently, PC-based BFN can be expressed as the solution of the following optimization problem:

(2) $$\begin{array}{l} min_{\text{C}}∥\text{C}-{\text{X}}^{T}\text{X}{∥}_{F}^{2} \end{array}$$

Where denotes the F-norm of a matrix. Such a reformulation of PC makes it easy to be explained under a unified graph learning framework as described in a recent study (Qiao et al., 2018).

### Sparse Representation

Different from PC that measures the full correlation between ROIs, SR is one of the statistical methods for modeling the partial correlation by regressing out the confounding effect from other ROIs (Peng et al., 2009). The model of SR is shown as follows:

(3) $$\begin{array}{l} min_{C_{ij}}\sum_{i=1}^{N}\left(∥{\text{x}}_{i}-\underset{j\neq i}{\sum }C_{ij}{\text{x}}_{j}{∥}^{2}+\lambda \underset{j\neq i}{\sum }|C_{ij}|\right), \end{array}$$

It can equivalently be expressed as the following matrix form:

(4) $$\begin{array}{l} min_{\text{C}}∥\text{X}-\text{X}\text{C}{∥}_{F}^{2}+\lambda {∥\text{C}∥}_{1}\\ \;s.t.C_{ii}=0,\;\forall i=1,\cdots \;,N, \end{array}$$

Where∥·∥_*F*_ and ∥·∥_1_ are *F*- and *L*_1_-norm of a matrix, respectively, and *C*_*ii*_ = 0 is employed for avoiding the trivial solution. Traditionally, $\text{X}-{\text{X}\text{C}}_{F}^{2}$ in Equation (4) is called a data-fitting term, and ∥**C**∥_1_ a matrix-regularized term. The balance between the two terms are controlled by a regularized parameter λ.

Note that the network matrix **C** obtained by solving Equation (4) is generally asymmetric. For facilitating comparison among different BFN estimation methods, we symmetrize it by $C_{ij}=sign\left(C_{ij}\right)\sqrt{C_{ij}C_{ji}}$, which is established on the theoretical basis for supporting the relationship between SR and partial correlation (Peng et al., 2009).

## BFN Estimation

### Motivation

As mentioned earlier, the estimated BFNs rely heavily on the quality of the data, but, unfortunately, the observed data in practice usually contain strong noises caused by many factors, especially, the head motion that results in both first- and higher-order effects (Bijsterbosch et al., 2017). The first-order effect mainly refers to the spatial misalignment from one volume to the next, which can be corrected by spatially registering each volume to a selected reference (Freire and Mangin, 2001; Dijk et al., 2012). In contrast, the higher-order effects, due to introducing noises into blood oxygen level dependent (BOLD) signals, are much harder to be removed from data and impact subsequent analyses even after the spatial registration (Power et al., 2012b; Murphy et al., 2013). Currently, three popular methods, including nuisance regression, independent component analysis (ICA), and scrubbing (Biswal et al., 1995; Rodriguez et al., 2012), are often employed to relieve such higher-order effects. In this paper, we mainly focus on the scrubbing operation, since it is closely related to our proposed method.

The scrubbing operation cleans some potentially “bad” volumes if the head motion, in the fMRI series, is greater than a threshold. Despite its simplicity, popularity and empirical effectiveness for removing “high-order” noises, such a scheme has the following drawbacks: (1) the amount of volumes removed by scrubbing is often relatively high [20–60% of all volumes (Bijsterbosch et al., 2017)], which would significantly reduce the statistical power of the data; (2) with a hard threshold, different numbers of volumes are generally removed across subjects, which leads to a bias or inconsistency in the estimated BFNs; (3) the scrubbing operation is independent of the ensuing BFN estimation task, and thus it is hard to know whether the remaining volumes absolutely benefit from BFN estimation, while the removed are necessarily unhelpful.

**Figure 1:**
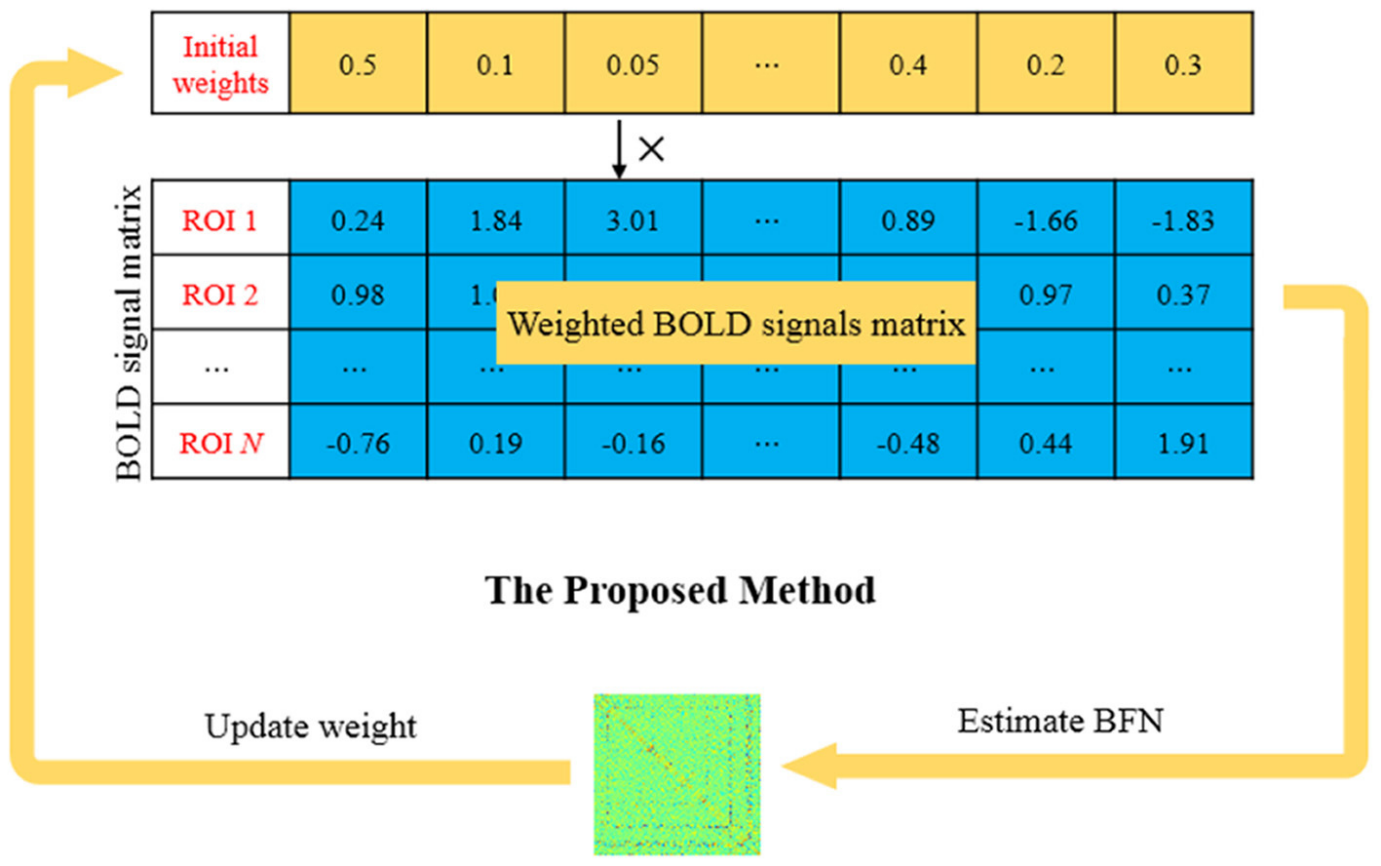
An example for intuitively illustrating how our proposed method works.

To address these issues, and differently from our previous work (Chen, 2019), we develop a new learning approach for BFN estimation based on weighted fMRI time series (instead of direct removal of volumes according to a hard threshold used in the traditional scrubbing scheme), where the weight put on each volume is adaptively estimated from the data. More specifically, as shown in Figure 1, an initial weight is first set for each time point (or volume) in the fMRI signals. Then, the following two steps are conducted till convergence: (1) a BFN is estimated based on the weighted fMRI signals, and (2) the weight on each volume is updated according to the estimated BFN. It is worth emphasizing that the two steps are not artificially designed by hand, but automatically derived from the proposed model that will be described in the next section.

### Proposed Model and Algorithm

In this paper, we develop a new learning framework that conducts BFN estimation and fMRI data “scrubbing” simultaneously in a single model as follows:

(5) $$\begin{array}{l} min_{C_{ij},W_{t}}\sum_{i,j=1}^{N}\sum_{t=1}^{T}T^{2}∥w_{t}{\text{x}}_{i}^{\left(t\right)}-\sum_{i\neq j}C_{ij}\left(w_{t}{\text{x}}_{j}^{\left(t\right)}\right){∥}^{2}\\ \;+\lambda \sum_{j\neq i}|C_{ij}|\\ s.t.\;0\leq w_{t}\leq 1,\sum_{t=1}^{T}w_{t}=1 \end{array}$$

where the *w*_*t*_ is the weight for the *t*th time point ${\text{x}}_{i}^{\left(t\right)}$ associated with the *i*th ROI, *C*_*ij*_ is the estimated functional connectivity between the *i*th and *j*th ROIs, and λ is a regularized parameter for balancing the two terms in the objective function. Compared with the traditional SR model given in Equation (3), the proposed model introduces a weight *w*_*t*_ into each time point in the fMRI signals, and the weight is optimized as a variable. Meanwhile, the proposed method implements new constraints of $0\leq w_{t}\leq 1,\sum_{t=1}^{T}w_{t}=1$, which not only avoids a trivial solution (i.e., *w*_*t*_ = 0, ∀*t*), but also gives a probability interpretation for the weight. Especially, Equation (5) would reduce to the traditional SR model if *w*_*t*_ = 1/*T* for all*t* = 1, 2, ⋯ , *T*, meaning that the proposed model is more flexible than SR.

Equivalently, Equation (5) can be expressed by the following matrix form:

(6) $$\begin{array}{l} \;min_{\text{C},\text{W}}{∥\text{W}\text{X}-\text{W}X\text{C}∥}_{F}^{2}+\lambda {\text{C}}_{1}\\ s.t.C_{ii}=0,0\leq w_{t}\leq 1,\sum_{t=1}^{T}w_{t}=1, \end{array}$$

Where **W** = diag(*w*_1_, *w*_2_, ···, *w*_*T*_) is a diagonal weight matrix, **C** is the adjacency matrix of BFN to be estimated, and **X** ∈ *R*^*T* × *N*^ is the fMRI data matrix (Without loss of generality, the data matrix **X** here has been re-defined as **X** = *T***X** for simplifying the expression).

### Comparison With Li et al.'s Method (Li et al., 2019a)

Our method actually follows Li et al.'s research line. Their model is listed as the following

$$\begin{array}{l} min_{\text{C},\text{V}}{∥\text{V}\text{X}-\text{V}X\text{C}∥}^{2}+\lambda ∥{\text{C}}_{1}∥-\gamma {∥\text{V}∥}_{1}\\ \;s.t.C_{ii}=0,0\leq v_{t}\leq 1,\forall i,t. \end{array}$$

**Figure 2:**
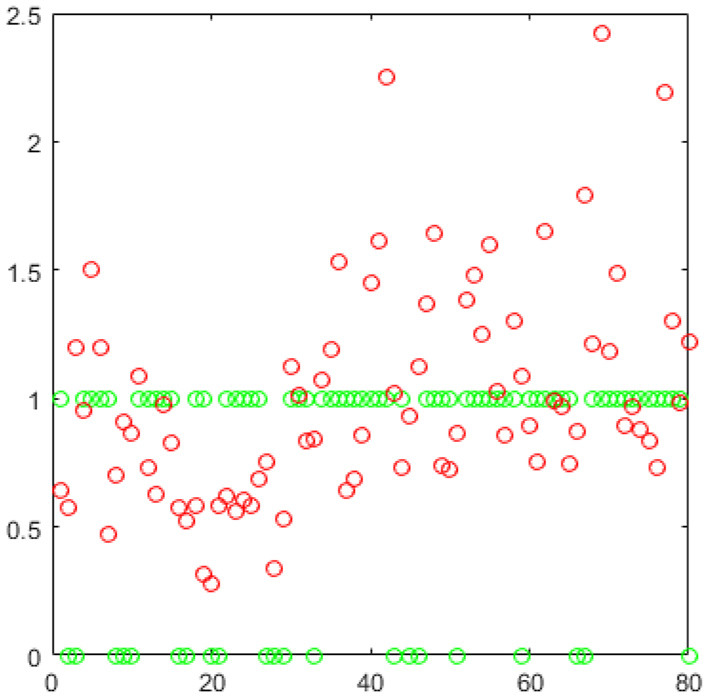
A comparison of two methods.

It is easy to see that these two approaches have something in common. Both of them construct the BFNs using a sparse representation technique and are task-dependent scrubbing methods by incorporating the scrubbing operation into the BFNs estimation task. It is also easy to see that there are some differences between them. In Li's model, they put a regularization term (W's l1 norm here) which can automatically find and remove the “bad” volumes. However, we put another new constraint as showed in (6) on the weights which leads to a more flexible and sufficient utilization of the volumes. The rationale behind this operation lies in that it is hard to ensure that the so called “bad” volumes are really unhelpful. Thus, Li's method may be viewed as a “hard” operation while ours a “soft” operation. Here we present an example as in Figure 2. These are the weights from the same sample under the same parameter, the green points are Li's and the red are ours. It seems interesting that they have certain coherence: the removed volumes also have smaller weights and the remaining have bigger weights. Further, In the last classification experiment, our performance is superior to Li's, which may verify our intuition that the “bad” volumes may indeed benefit our BFNs construction task.

### Optimized Algorithm

In consideration of the fact that two variables, **C** and **W**, are involved in **Equation (6)**, we employ the alternative optimization (AO) (Bazaraa et al., 2013) to solve this problem as given in the two following steps.

**Step 1**: with a fixed **W**, estimate BFN **C**.

Note that the objective function in Equation (6) is non-differentiable due to the *L*_1_-norm regularizer. Therefore, we solve it *via* the proximal method (Combettes and Pesquet, 2011). Specifically, for the data-fitting term $f\left(\text{X},\text{C}\right)={∥\text{W}\text{X}-\text{W}X\text{C}∥}_{F}^{2}$, we first calculate its gradient w.r.t. **C** and have $\nabla_{\text{C}}f\left(\text{X},\text{C}\right)=2\left({\text{X}}^{T}{\text{W}}^{T}\text{W}X\text{C}-{\text{X}}^{T}{\text{W}}^{T}\text{W}\text{X}\right)$. As a result, we have the following updated formula for **C** according to the gradient descent criterion:

(7) $$\begin{array}{l} C_{k}=C_{k-1}-\alpha_{k}\nabla_{C}f\left(\text{X},C_{k-1}\right), \end{array}$$

Where α_*k*_ denotes the step size of the gradient descent. Then, the current **C** is mapped into a “feasible region” by the following proximal operator (Combettes and Pesquet, 2011):

(8) $$\begin{array}{l} \text{pro}{\text{x}}_{\lambda {∥\cdot ∥}_{1}}\left(\text{C}\right)={\left[sgn\left(C_{ij}\right)\times \text{max}\left(abs\left(C_{ij}\right)-\lambda ,0\right)\right]}_{N\times N} \end{array}$$

Where *sgn*(*C*_*ij*_) and *abs*(*C*_*ij*_) return the sign and absolute value of *C*_*ij*_, respectively.

**Step 2**: with a fixed **C**, update **W**.

When **C** is a constant matrix, Equation (6) reduces to the following optimization problem:

(9) $$\begin{array}{l} \;min_{\text{W}}\;{∥\text{W}\text{X}-\text{W}X\text{C}∥}_{F}^{2}\\ s.t.0\leq w_{t}\leq 1,\sum_{t=1}^{T}w_{t}=1. \end{array}$$

We can rewrite it as follows:

(10) $$\begin{array}{l} min_{W_{t}}\;\sum_{t=1}^{T}∥{\text{X}}^{\left(t\right)}-{\text{X}}^{\left(t\right)}\text{C}{∥}^{2}w_{t}^{2}\\ \;s.t.0\leq w_{t}\leq 1,\;\sum_{t=1}^{T}w_{t}=1 \end{array}$$

By defining the Lagrange multipliers of Equation (10), we have

(11) $$\begin{array}{l} L\left(\text{W},\alpha \right)=\sum_{t=1}^{T}∥{\text{X}}^{\left(t\right)}-{\text{X}}^{\left(t\right)}\text{C}{∥}^{2}w_{t}^{2}-\alpha \left(\sum_{t=1}^{T}w_{t}-1\right) \end{array}$$

Let the derivative of Equation (11) w.r.t. **W** be zero, that is,

(12) $$\begin{array}{l} 2∥{\text{X}}^{\left(t\right)}-{\text{X}}^{\left(t\right)}{\text{C}∥}^{2}w_{t}-\alpha =0, \end{array}$$

And then, we get

(13) $$\begin{array}{l} w_{t}=\frac{1}{2}\alpha ∥{\text{X}}^{\left(t\right)}-{\text{X}}^{\left(t\right)}{\text{C}∥}^{-2}. \end{array}$$

Due to the constraint $\overset{T}{\underset{t=1}{\sum }}w_{t}=1$, we have the following equation:

(14) $$\begin{array}{l} \sum_{t=1}^{T}\alpha {∥{\text{X}}^{\left(t\right)}-{\text{X}}^{\left(t\right)}\text{C}∥}^{-2}=2, \end{array}$$

Which results in the following solution of α,

(15) $$\begin{array}{l} \alpha =2/\sum_{t=1}^{T}∥{\text{X}}^{\left(t\right)}-{\text{X}}^{\left(t\right)}{\text{C}∥}^{-2} \end{array}$$

Finally, we substitute **Equation (15)** into **Equation (13)**, and get:

(16) $$\begin{array}{l} w_{t}=∥{\text{X}}^{\left(t\right)}-{\text{X}}^{\left(t\right)}\text{C}{∥}^{-2}/\sum_{t=1}^{T}∥{\text{X}}^{\left(t\right)}-{\text{X}}^{\left(t\right)}{\text{C}∥}^{-2}. \end{array}$$

This gives a closed-form solution for *w*_*t*_ with a clear physical explanation that the weight of the *t*th time point depends on the data fitting error at current moment. In other words, the weights tend to be related to the quality of data.

Now, we simply summarize the above algorithm in the following Table 1.

**Table 1:** Algorithm.

Input: **X**, λ //data and parameters
Output: **C**, **W** //brain network and weighted matrix
Initialize **W**;
while not converge
while not converge
**C**←**C**−α∇_**C**_*f*(**X**, **C**);
*C*←*prox*_λ_∥·∥__1__(*C*) = [*sgn*(_*C*_*ij*_) × max(*abs*(*C*_*ij*_)λ, 0)]*N* × *N*_;
end
update **W** by Equation (17);
end
return **C**, **W**;

## Experiments

We will evaluate the performance of our method by applying it to the early identification of AD. As a common form of dementia, AD occurs most frequently in the aged population. It not only influences the daily life of patients, but also causes heavy economic burden for the patient's family and society (Peng et al., 2019). As an intermediate between AD and normal aging, MCI is believed to be the earliest clinically detectable stage of progression toward AD (Li et al., 2020). Every year, subjects with MCI may evolve to AD with a rate of 10–15% (Petersen and Roberts, 2009), while healthy controls develop into dementia with a rate of 1–2% annually (Bischkopf et al., 2010). Therefore, the early detection of MCI is vital for delaying the transition from MCI to AD (Chaves et al., 2012; Zhang et al., 2019). In the next experiments, we estimate BFNs based on fMRI data and apply the estimated BFNs as features to classify the subjects with MCI from NCs.

### Data Acquisition and Preprocessing

The data used in this study were shared by a recent study (Yan et al., 2013), and can be freely downloaded from Neuroimaging Informatics Tools and Resources Clearinghouse (NITRC).^1^ Concretely, 91 subjects (45 MCIs and 46 NCs) were participated in our experiment. The subjects were scanned by 3.0T Siemens scanners with the following scanning parameters: TR/TE is 3,000/30 ms, imaging matrix size is 74 × 74, 45 slices, and voxel size is 2.97 × 2.97 × 3 mm^3^.

The acquired fMRI images were processed by SPM8 toolbox^2^ based on the well-accepted pipeline (Rubinov and Sporns, 2010). For each subject, the first 10 volumes were removed for signal stabilization. The remaining volumes were first corrected for different slice acquisition timing and head motion. Then, regression of ventricle and white matter signals as well as six head-motion profiles were conducted to reduce the influences of nuisance signals. The fMRI series were further band-pass filtered from 0.01 to 0.08 Hz. After that, the corrected images were registered to Montreal Neurological Institute (MNI) standard space, and, based on the automated anatomical labeling (AAL) atlas (Tzourio-Mazoyer et al., 2002), pre-processed fMRI signals were partitioned into 90 ROIs. Please refer to (Qiao et al., 2016) for the description of the preprocessing pipeline in detail. Finally, for each subject, the mean time series of each ROI (Michel et al., 2012) were extracted and put into the column of a data matrix **X** that is the only material for estimating BFNs.

### Brain Functional Network Estimation

As mentioned above, now each subject corresponds to a data matrix, by which we can construct BFNs using different methods. For SR-based methods, they involve a regularized parameter λ that generally affects the subsequent classification performance significantly. Therefore, in our experiments, we select the optimal parametric values for them based on the training set from a candidate range of [2^−5^, 2^−4^, ⋯ , 2^0^, ⋯ , 2^4^, 2^5^]. Contrary to SR, the PC-based BFN estimation model is parameter-free. However, to improve its flexibility and conduct a fair comparison, we introduce a threshold parameter in PC to remove a proportion of weak edge weights (connections) in the estimated BFN. To be consistent with SR-based methods, we also use 11 candidate values ranging from [1%, 10%,⋯ , 90%, 100%], where the percentage means the proportion of weak connections that are discarded.

### Feature Selection and Classification

With the constructed BFNs for all subjects, the next tasks are feature selection and classification. In this paper, we regard the pairwise functional connections between 90 ROIs as features for MCI identification. As a result, the symmetric BFN adjacent matrix contains 90 × (90 − 1)/2 = 4005 features, which would lead to the so-called curse of dimensionality and overfit the training data. Therefore, we reduce the data dimensions by selecting discriminative features based on the *t*-test with fixed *p*-values prior to the subsequent classification task. In particular, we set *p*-value = 0.005 for PC, and 0.01 for other methods, due to their empirically best performance. Based on the selected features, we use the popular linear SVM with default parameter (C = 1) for classification.

**Figure 3:**
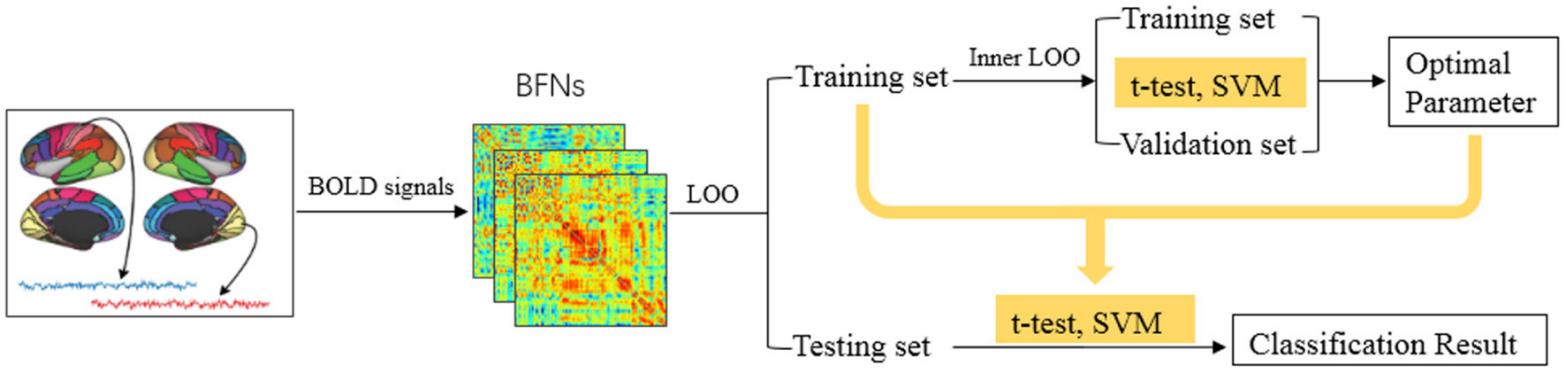
The MCI identification pipeline based on the estimated BFNs.

The detailed pipeline for feature selection and classification is shown in Figure 3. Due to the small sample size, we employ the leave-one-out (LOO) cross validation strategy for testing the performance of the involved methods. Specifically, for a total of *S* subjects, one is left out in each loop for testing, while the remaining *S-1* subjects are used for training the model.

As described earlier, the free parameter involved in the BFN estimation methods may have a significant influence on the network topology and the final classification result. Therefore, for each method we first estimate BFNs based on different parametric values, and then in each loop we use an inner LOO to validate the classification accuracy on the training data for finding the optimal parametric values in the range of candidate set. Concretely, in each iteration, one of the *S-1* training samples/subjects is left out to validate the performance, while the remaining *S-2* subjects are for training the model. Then, with the optimal network parametric value, we re-run feature selection and SVM classifier. The final classification accuracy is calculated by averaging the results from all subjects.

### Results

#### Brain Network Visualization

**Figure 4:**
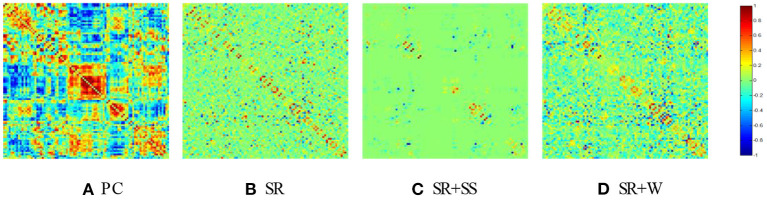
The BFN adjacency matrices constructed by different methods. **(A)** PC, **(B)** SR, **(C)** SR+SS, **(D)** SR+W.

In our experiment, we construct BFNs using four different methods, including PC, SR, SR+SS (Li et al., 2019a) and the proposed adaptively-weighted scheme (namely SR+W). The SR-based models in this paper are solved via SLEP toolbox (Liu et al., 2011). The adjacency matrices of BFNs estimated by different methods are shown in Figure 4. It can be observed that the BFN estimated by PC (a) has a topology that is highly different from those estimated by SR-based methods, since they use different data fitting terms (or, equivalently, capture different statistical dependency between ROIs). In contrast, the SR-based methods lead to similar topological structures since using the same kind of data fitting term for capturing the partial correlation. Compared with the traditional SR, SR+SS (c) affect the network structure significantly due to the direct and stringent removal of volumes from fMRI time series, while the proposed SR+W method (d) can preserve the original network structure well by using a more flexible and lenient volume-weighted strategy.

#### Classification Performance

In this paper, we use four quantitative measures, including accuracy (ACC), sensitivity (SEN), specificity (SPE), and false positive rate (FPR) to evaluate the classification performance of different methods. Their mathematical definitions are given as follows:

$$\begin{array}{l} \text{ACC}=\frac{TP+TN}{TP+FP+TN+FN}\\ \text{SEN}=\frac{TP}{TP+FN}\\ \text{SPE}=\frac{TN}{TN+FP}\\ \text{FPR}=\;\frac{FP}{FP+TN} \end{array}$$

Where *TP, TN, FP*, and *FN* indicate true positive, true negative, false positive, and false negative, respectively. In this paper, we treat the MCI samples as positive class and the NC samples as negative class.

**Figure 5:**
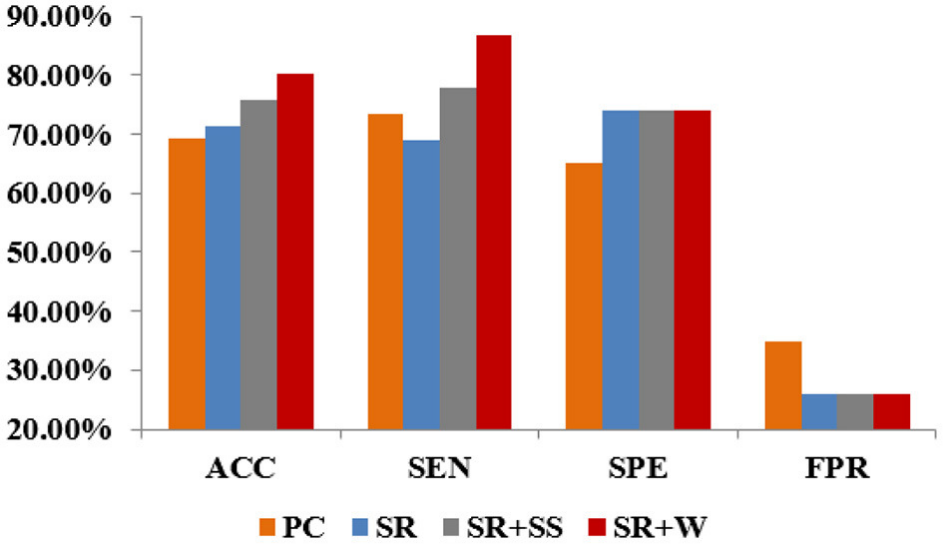
Classification results corresponding to different methods.

The classification results corresponding to different methods are reported in Figure 5. Despite its popularity, PC-based BFNs tend to involve many indirect and redundant connections (as shown in Figure 4 earlier), which may affect its final classification performance. In contrast, SR can regress out the confounding effect from other ROIs, and thus achieve a better classification accuracy than PC. Different from the traditional PC and SR, SR+SS can detect and remove some potential “dirty” data, which may be one of the reasons for the improved classification accuracy. The proposed SR+W method further introduces a more flexible strategy into the traditional SR model for adaptively weighting the fMRI data, and achieves the best classification accuracy, illustrating that the adaptively weighted scheme can improve the quality (at least, the discriminative power) of the estimated BFNs to some extent.

#### Sensitivity to Network Model Parameters

**Figure 6:**
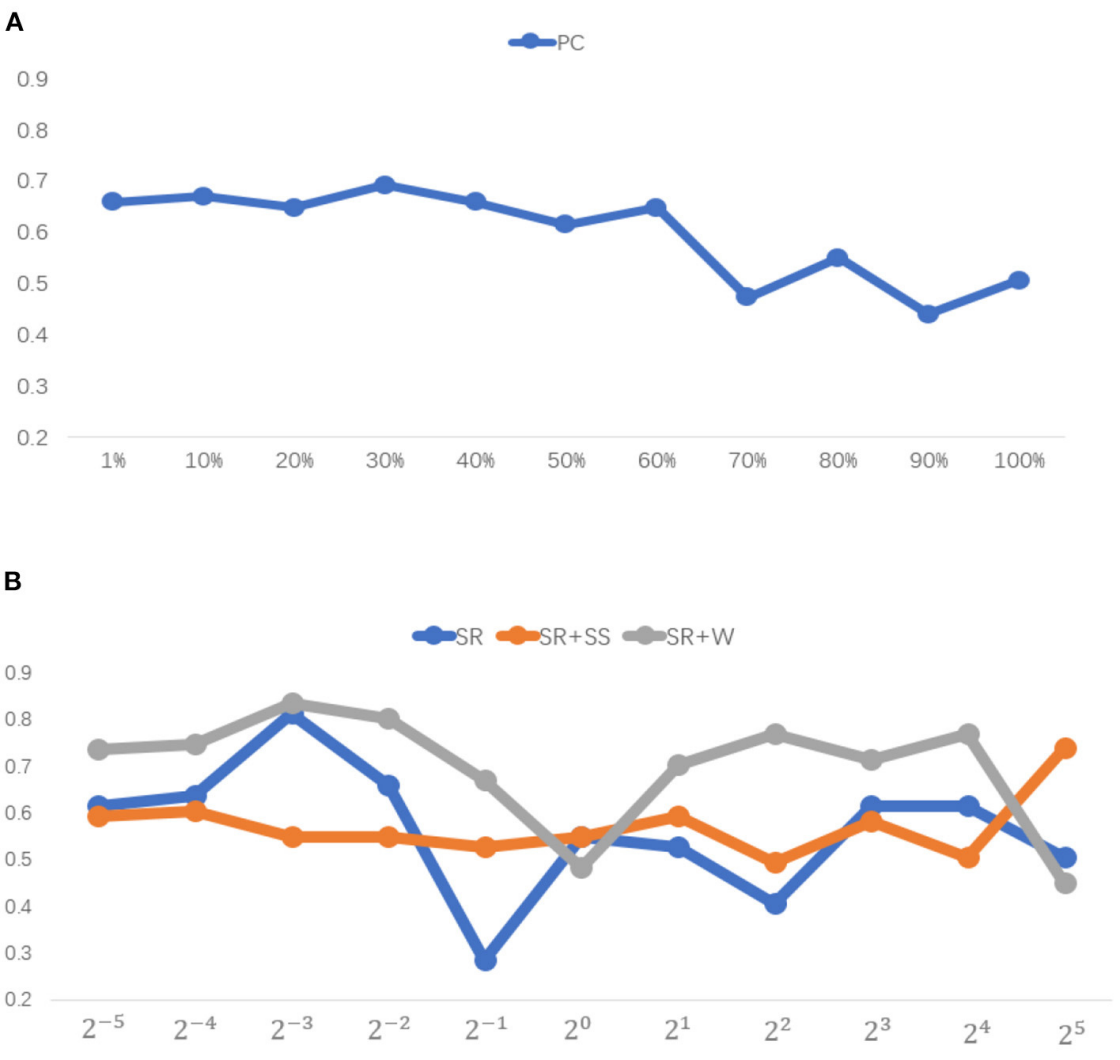
Classification accuracy associated with different method under 11 different parametric values.

In general, model parameters have a heavy influence on the network topology and then the final classification accuracy. To investigate the sensitivity of the involved methods to different values of parameters, we conduct classification experiments on the above data set via LOO cross validation. We report the classification accuracy corresponding to different parametric values in Figure 6 for different BFN estimation methods. It can be observed that the proposed method achieves the best performance for most of the parametric values. However, it is exceedingly sensitive to the model parameter.

#### Top Discriminative Features (Networks Connections)

**Figure 7:**
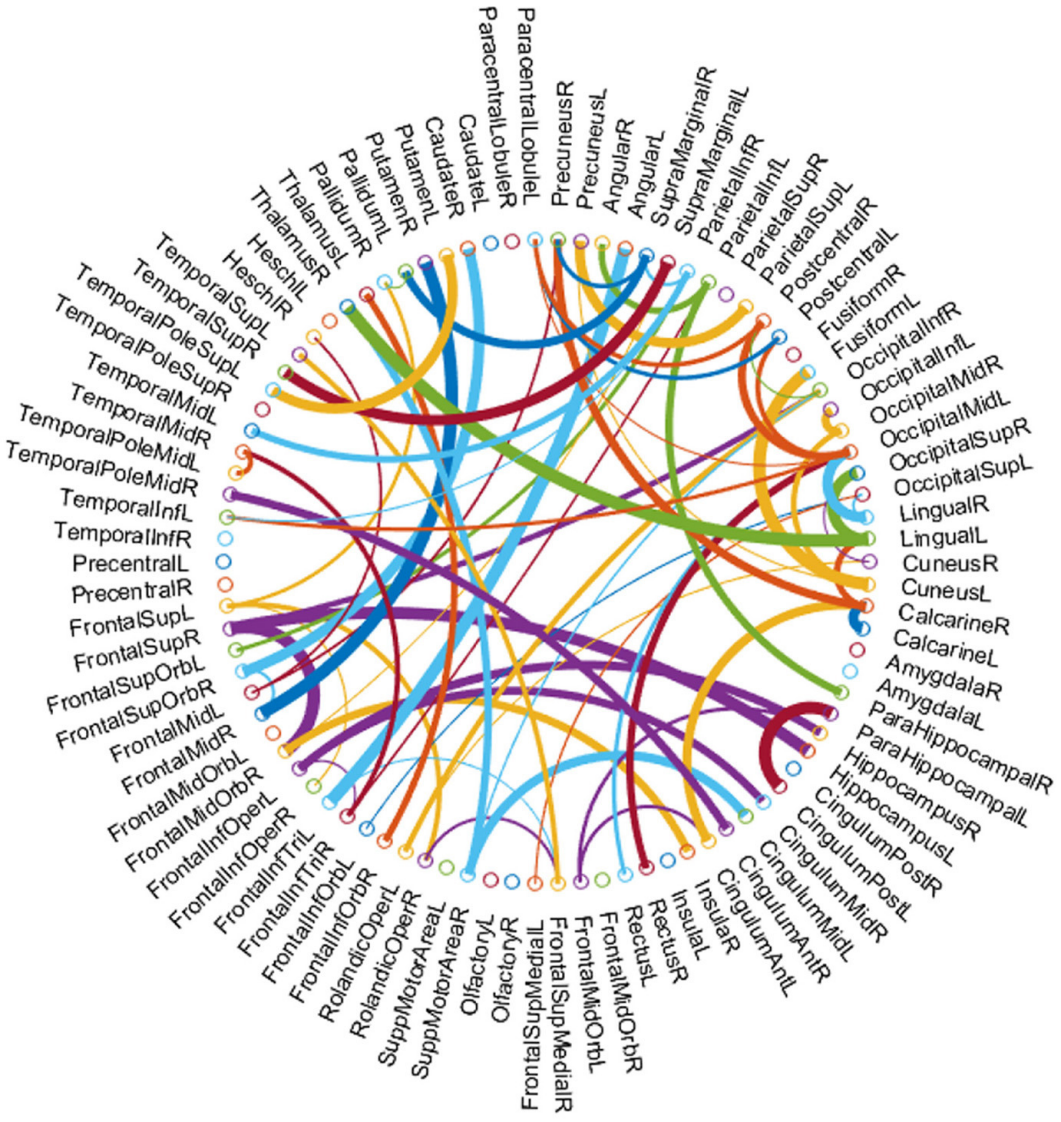
The most discriminative features (connections) for the 90 ROIs of AAL template involved in the classification tasks. The figure is created by circularGraph tool, shared by Paul Kassebaum. http://www.mathworks.com/matlabcentral/fileexchange/48576-circulargraph.

In this paper, we regard the connections between ROIs as features for identifying subjects with MCI from NCs in the dataset. Since the selected features (network connections) may be different in each folder of cross validation, we sort features according to their average of *p*-values on all folders. As a result, by recording the selected features during the training process, we select 69 discriminative connections and visualize them in Figure 7. Note that the thickness of an arc is inversely proportional to the *p*-values for indicating the discriminative power of a connection in the classification task. The colors of arcs are randomly generated only for a clearer visualization. From Figure 7, we can find that the brain regions associated with top discriminative features include the temporal gyrus, parietal lobules, parahippocampus, supramarginal gyrus and precuneus, etc. This result conforms to the previous neuroimaging biomarker reports and the pathology studies on MCI (Greicius et al., 2007; Albert et al., 2011). The existing studies (Wang et al., 2013; Gardini et al., 2015) have pointed out that patients with MCI and AD have the same regional network connectivity anomalies compared with healthy controls. Therefore, it has practical significance to realize the early diagnosis of MCI and inhibit its evolution into AD.

#### Performance Evaluation on Simulated Data

**Figure 8:**
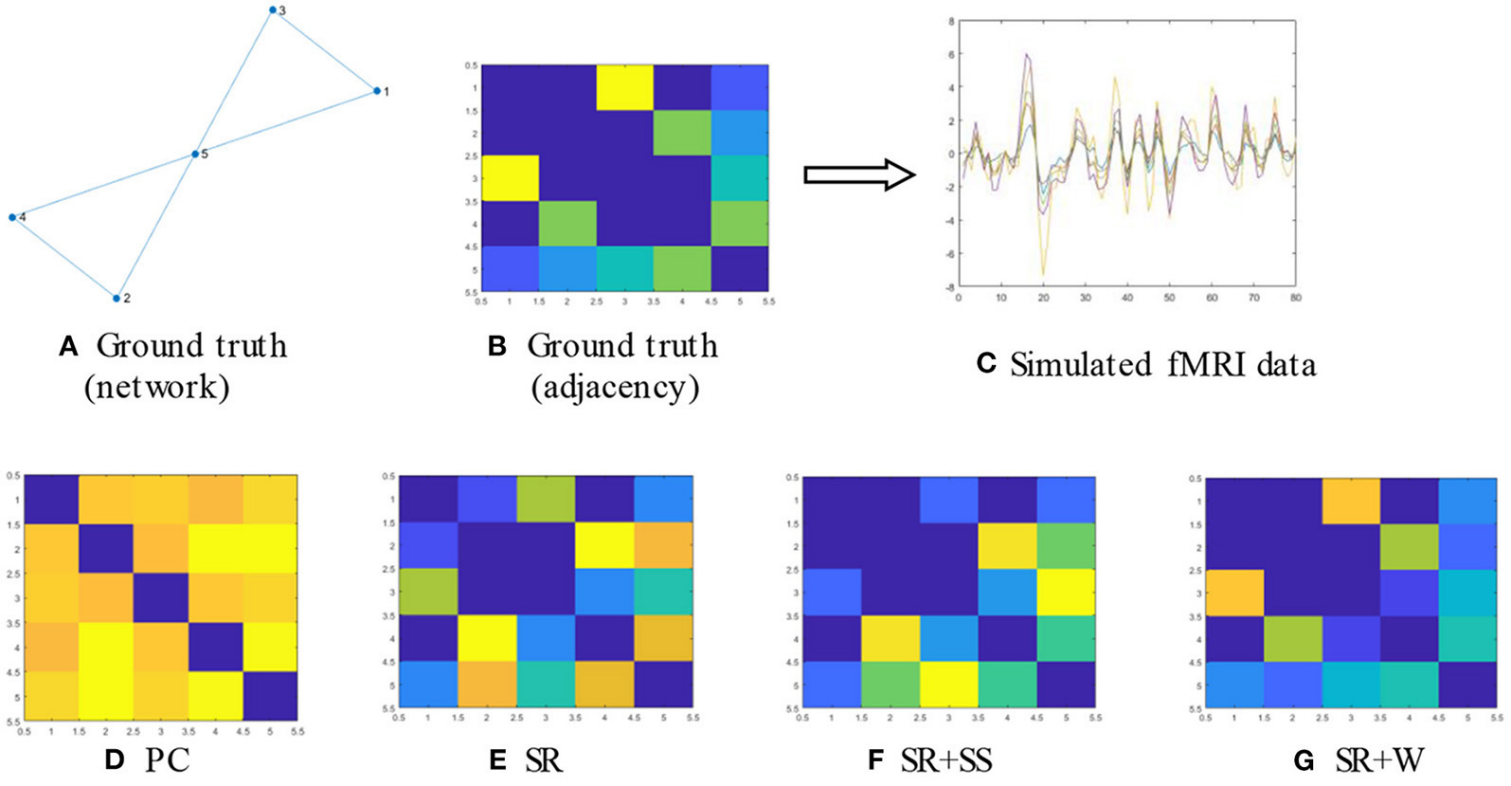
**(A,B)** The given network as ground truth; **(C)** The simulated BOLD signals generated according to our motivation; **(D–G)** The estimated FBNs by running four different methods based on the simulated BOLD signals in **(C)**.

We also carried out experiments on a set of simulated Bold signals for evaluating the generalizability of the proposed algorithm and analyzing their ability to detect the true network. At first, we prepared a known network as Figures 8A,B beforehand which is consistent with the hypothesis of our model as the ground truth, that is, it has a clear sparse structure. Then, based on this ground truth network we generate the Bold signals with 80 time points using linear sparse combinations of two given real ROI time series *x*_1_, *x*_2_, associated with ROI1 and ROI2, that is

(17) $$\begin{array}{l} data=\left[x_{1},x_{2}\right]\left[\begin{array}{ccccc} 1 & 0 & 3 & 0 & 1\\ 0 & 1 & 0 & 2 & 0.5 \end{array}\right] \end{array}$$

Further, to be more in line with the actual scenario, we change several time points randomly in the generated data for simulating the possible artifacts, noises, or “abnormal” resting-state processes. Specifically, we add Gaussian noise to the data to simulate the inevitable noise in real settings and introduce five “dirty” points to simulate the possible functional “noises,” by setting them off the main direction. The eventually signals are illustrated as in Figure 8C. Finally, we estimate FBNs based on the simulated BOLD signals using different methods, and visualize the estimated results in Figures 8D–G.

We have the following observation from Figure 8: (1) PC leads to some false connections and produce a dense topology; (2) SR cannot recover the original graph yet as PC although it can remove some connections which makes it exhibit a little cleaner structure. (3) SR+SS can basically reflect the original graph except that an extra edge has been added. However, the order of the weight values have been significantly altered. (4) Different from the above three methods, the proposed algorithm can not only reveal the original topology structure but also preserve the order of the weight values even if it also has an additional edge. The similarity between the estimated brain networks and ground truth by Pearson's correlation coefficient is shown in Table 2. The results show that our method achieves the highest similarity to the ground truth.

**Table 2:** The similarity between ground truth network and the generated FBNs.

	**PC**	**SR**	**SR+SS**	**SR+W**
Ground truth	33.4%	49.1%	62.7%	78.6%

## Conclusion

It is known that the quality of estimated BFNs heavily depends on the observed data. However, in practice, the observed fMRI data are commonly influenced by many factors, especially head motion. Although complex preprocessing pipelines are employed to remove the effects of head motion as much as possible, they are fully independent to the ensuing BFN estimation. Therefore, in this paper we propose a new learning model to estimate BFN together with data “scrubbing” by adaptively weighting the volumes in fMRI time series. Then, we develop an efficient AO algorithm to solve the proposed model and get a closed-form weight update formula with a clear physical explanation. Experiments on a publicly available dataset show that our estimated BFN can result in the best classification accuracy for an MCI identification task. Finally, but interestingly, it is worth emphasizing that, despite its helpfulness in the final performance, the proposed BFN estimation method is unsupervised, meaning that both the weights on data and the network itself are learnt without using the label information. Regarding the fact that our final goal is to improve the classification accuracy, in the future we therefore plan to develop supervised BFN learning algorithms toward a better (at least more discriminative) human brain connectome.

## Data Availability Statement

The datasets presented in this study can be found in online repositories. The names of the repository/repositories and accession number(s) can be found at: http://www.nitrc.org/projects/modularbrain/, Neuroimaging Informatics Tools and Resources Clearinghouse.

## Ethics Statement

The studies involving human participants were reviewed and approved by Geneva University Hospital. The patients/participants provided their written informed consent to participate in this study.

## Author Contributions

All authors listed have made a substantial, direct and intellectual contribution to the work, and approved it for publication.

## Conflict of Interest

DS was employed by company of Shanghai United Imaging Intelligence. The remaining authors declare that the research was conducted in the absence of any commercial or financial relationships that could be construed as a potential conflict of interest.

## Footnotes

^1^http://www.nitrc.org/projects/modularbrain/
